# Integrated AI and DFT: A Revolutionary Computational Breakthrough for Microwave‐Absorbing Materials Design

**DOI:** 10.1002/advs.202513098

**Published:** 2025-10-08

**Authors:** Shengchong Hui, Lechun Deng, Limin Zhang, Hao Shen, Qiang Chen, Hongjing Wu

**Affiliations:** ^1^ MOE Key Laboratory of Material Physics and Chemistry under Extraordinary Conditions School of Physical Science and Technology Northwestern Polytechnical University Xi'an 710072 P. R. China; ^2^ State Key Laboratory of Solidification Processing School of Materials Science and Engineering Northwestern Polytechnical University Xi'an 710072 P. R. China; ^3^ Department of Applied Physics School of Science Chang'an University Xi'an 710064 P. R. China

**Keywords:** artificial intelligence, density functional theory, microwave‐absorbing materials

## Abstract

Microwave‐absorbing materials (MAM) are important for modern technologies, but the design of MAM remains hindered by insufficient experimental characterization of the microscopic mechanisms governing electromagnetic (EM) energy dissipation. While Density Functional Theory (DFT) provides theoretical evidence for probing electronic structures, its application faces significant challenges. These include discrepancies between theoretical models and realistic structures, inadequate treatment of alternating EM fields, and errors in strongly correlated systems. Recent advances in Artificial Intelligence (AI) offer transformative opportunities to address these challenges. AI algorithms can predict and model electronic responses under physical equation constraints, accelerate the screening of computational parameters, and enhance the reliability of DFT‐based interpretations. This perspective critically illustrates the current state of DFT applications and the limitations of existing approaches in MAM, while analyzing contemporary strategies to mitigate DFT limitations. In addition, it is proposed prospectively that future research should integrate physics‐informed neural networks, adaptive algorithms, and DFT to address the current dilemma. This not only emphasizes the transformative potential of AI but also unlocks scalable design principles for the next generation of MAM.

## Graphical abstract

This perspective examines DFT in microwave‐absorbing materials, highlighting challenges like atomic configuration inaccuracies and electromagnetic field simulation limitations. It proposes integrating DFT and AI techniques such as active learning, Physical Information Neural Networks, and Graph Neural Networks for precise electron dynamics modeling and calculation, enabling rational design of high‐performance microwave‐absorbing materials with improved accuracy and scalability.

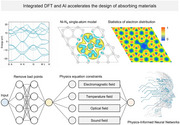

## 1 Introduction

Microwave‐absorbing materials (MAM) play an irreplaceable role in military equipment, electronic devices, and 5G communications, owing to their core functions of interference reduction and stealth.^[^ ^1^, ^2^, ^3^, ^4^, ^5^ ^]^ The microwave attenuation performance of these materials is fundamentally governed by microscopic physical processes under electromagnetic (EM) field excitation, which involve multiple energy dissipation mechanisms.^[^ ^6^ ^]^ The conventional research paradigm mainly relies on experimental characterization of macroscopic parameters (e.g., permittivity, permeability, conductivity, and phase composition), while empirically inferring the microscopic loss mechanisms. However, this approach faces a critical limitation: the lack of direct experimental evidence to confirm the actual occurrence of specific loss mechanisms within the material. However, existing characterization techniques remain inadequate for in situ observation of microscopic processes such as carrier dynamics, localized charge transfer processes, and polarization behavior. This stems from the spatial and temporal resolution constraints of conventional characterization techniques, which fail to capture transient electronic states during EM field‐matter interactions, thereby restricting the design and development of high‐performance MAM.

In this context, first‐principles calculations based on density functional theory (DFT) offer a promising analytical framework for addressing the challenges described above. By solving the ground‐state electronic structure of quantum systems, this methodology allows the investigation of electron dynamic response characteristics within a quantum mechanical statistical framework at the atomic scale, thereby revealing the microscopic origins of EM energy dissipation and presenting it in visual data. So far, DFT calculations have been extensively employed in the investigation of MAM for the vast majority of systems, such as carbon‐based materials, ferrite, boride, sulfide, etc., and the computational outcomes provide critical theoretical supports for understanding the EM response mechanisms.^[^ ^7^, ^8^, ^9^, ^10^, ^11^ ^]^ Nevertheless, the application of DFT in wave absorption still faces theoretical and methodological challenges. These include the deviation of atomic configurations from real materials; the inability to calculate electronic states under alternating EM fields; and the computational errors of generalized functions in strongly correlated systems. These problems lead to the insufficient reliability of DFT when interpreting microscopic mechanisms in specific systems, thereby impeding its advancement in designing novel MAM.

Recent advances in Artificial Intelligence (AI) offer promising strategies to address the above limitations.^[^ ^12^ ^]^ Specifically, active learning (AL) strategies applied to deep neural networks hold promise for optimizing current computational modeling methods. By training on highly informative DFT data, micro‐scale electron dynamics simulations can be performed with near‐real accuracy, thereby modeling actual atomic configurations with defects and interfaces. AI‐driven Physical Information Neural Networks (PINNs) can be trained on datasets incorporating physical field constraints and material properties. By learning complex structure‐property relationships, they can predict dynamic electronic response without explicitly solving computationally complex time‐dependent Kohn–Sham equations. In strongly correlated systems where standard DFT functionals fail, graph neural networks (GNNs) can transmit atomic information in graphical form, thereby directly establishing correlations between atomic configurations and electronic properties. Bayesian optimization techniques transform empirical parameter selection into data‐driven optimization problems. When combined with neural networks trained on high‐throughput datasets, they enable rapid screening of DFT parameter configurations to achieve optimal computational outputs. These approaches not only can alleviate current computational bottlenecks but also can facilitate a paradigm shift in research that enables the rational design of next‐generation MAM with higher accuracy and scalability.

This perspective provides a comprehensive summary of the current state of DFT applications in the microwave absorption field. It specifically examines representative computational outputs and their established links to material characteristics, with the objective of formulating a guideline for interpreting the DFT calculation results in MAM. It also critically points out the inherent limitations encountered when applying DFT to specific systems and outlines contemporary approaches to alleviate these challenges. Finally, this perspective elucidates the advantages of integrating AI with DFT calculations and prospectively discusses how future AI‐DFT integration could accelerate the research of MAM.

## 2 The Correlation Between DFT Outputs and Material Properties

To guide the analysis of DFT calculations for MAM, the following content will systematically elucidate the correlation between the typical outputs derived from DFT calculations and the material parameters that regulate microwave absorption performance.

### 2.1 Predicting the Conductive Properties of MAM by Band Structure

**Figure 1:**
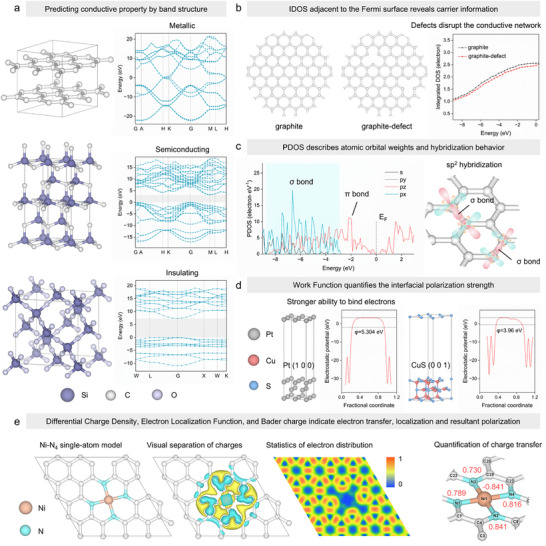
a) The band structures of graphite, SiC, and SiO_2_; b) The IDOS curves of graphite and graphite with defects; c) The PDOS images of single‐layer graphite model; d) The calculated WF of Pt (1 0 0) and Cu (0 0 1) models with 20 Å vacuum layer; e) The calculated DCD, ELF, and Bader charge of Ni‐N_4_ single‐atom model.

In the field of microwave absorption research, band structures derived from DFT calculations provide fundamental insights into the microscopic origins of dielectric properties. Specifically, band structure analysis focuses on the bandgap width, which directly governs carrier transport behavior and determines the conductivity (metallic, semiconducting, or insulating) of materials.^[^ ^13^ ^]^ As shown in **Figure** **1**a, the band structures of graphite, SiC, and SiO_2_ show that these materials possess metallic, semiconducting, and insulating properties, respectively. By analyzing the bandgap width characteristics, researchers can qualitatively evaluate the conduction loss in MAM. This is because the zero‐bandgap or narrow‐bandgap materials typically exhibit higher intrinsic conductivity compared to wide‐bandgap materials, leading to correspondingly stronger conduction loss. For example, researchers have demonstrated that In‐doped MoS_2_ possesses a narrower bandgap (0.02 eV) and higher conductivity loss compared to pure MoS_2_ (2.09 eV).^[^ ^14^ ^]^

It is worth noting that in DFT calculations, classifying materials as semiconductors or conductors cannot rely solely on band structure diagrams. This is because the band structures of some practical narrow‐gap semiconductors (e.g., 1T‐MoS_2_) paradoxically exhibit metallic bandwidth characteristics in DFT calculations.^[^ ^15^ ^]^ The bandgaps of some actual conductors (e.g., graphene structure with defects) may also be computationally overestimated, leading to their misclassification as narrow‐gap semiconductors. Furthermore, the choice of computational functional significantly impacts the calculated results of band structures. For instance, the Local Density Approximation (LDA) and Generalized Gradient Approximation (GGA) often systematically underestimate bandgap values, with deviations reaching 40% or more compared to experimental values. In contrast, hybrid functionals such as HSE06 and B3LYP effectively correct this error, typically yielding bandgap results that closely match experimental values. We demonstrate that the choice of functional essentially reflects a trade‐off between computational efficiency and accuracy, requiring researchers to select an appropriate functional type based on their specific requirements.

### 2.2 Density of States (DOS) Reveals Carrier Concentration, Atomic Orbital Weights, and Hybridization Characteristics

Density of states (DOS) curves provide two types of critical information for microwave absorption research. The first is the integrated DOS (IDOS) near the Fermi level, which offers an estimate of the total number of electrons and serves as a semi‐quantitative measure of the electron filling state. Furthermore, the IDOS within a specific energy window around the Fermi level corresponds to the electron density available for electrical conduction, which directly influences the effective carrier concentration.^[^ ^16^ ^]^ Therefore, by comprehensively analyzing the IDOS, researchers can gain a qualitative understanding of the conductive behavior of materials. Especially in the zero‐bandgap system, IDOS can serve as an indirect reference for developing an assessment framework of conductivity. Figure 1b demonstrates IDOS curves of graphite and graphite with defects, indicating that the defects can disrupt the conductive network, leading to a decrease in conductivity. The researchers also demonstrated that growing Mo single atoms in the MoS_2_ system significantly increases its conductivity, which was supported by IDOS.^[^ ^17^ ^]^

The second is the projected DOS (PDOS), which enables the assessment of contribution weights from distinct atomic orbitals within the total DOS, such as the *p* orbitals of non‐metallic elements and the *d* orbitals of magnetic transition metals. Furthermore, the presence of overlapping peaks in the PDOS spectra of two elements suggests hybridization between their corresponding atomic orbitals, a key factor in microwave energy dissipation. For example, sp^2^ hybridization of carbon in graphene facilitates conjugated π‐bonds that promote electron localization (Figure 1c), while sp^3^ hybridization can modify defect or doping characteristics. In metal‐organic frameworks (MOFs), hybridization between metal nodes and organic linkers modulates the spatial electron distribution, and in ferrites, hybridization of Fe^3^⁺ with oxygen influences magnetic anisotropy.^[^ ^18^ ^]^ By resolving the spatial distribution of electronic filling states and orbital hybridization characteristics through PDOS, researchers can reveal the extent to which specific elements influence conductivity and magnetic properties. For example, researchers have calculated the PDOS of the Er‐N_4_ model and shown that the enhanced conduction loss is mainly due to the contribution of the *f*‐orbital and *p*‐orbital electrons of the Er atom.^[^ ^19^ ^]^

### 2.3 Describing Interfacial Polarization Intensity through Work Function (WF)

The differences in surface atomic exposure types, atomic packing densities, and symmetry characteristics among distinct crystal planes lead to the differential formation of surface potential, resulting in varying degrees of electron confinement. This electron binding strength can be quantitatively described using DFT‐based WF calculations, and the higher WF corresponds to stronger electron confinement on the corresponding crystal planes. For example, the calculated WF of Pt (1 0 0) and Cu (0 0 1) models shows that Pt (1 0 0) facet possesses the stronger ability to bind electrons (Figure 1d). Typically, the minimal variation in WF between homogeneous crystalline planes results in relatively uniform constraining forces on electrons during transport across grain boundaries, which enables efficient interfacial electron transport between adjacent crystal planes. The lack of electron accumulation at the interface makes it difficult for interfacial polarization to occur within single‐phase crystals. Compared to single‐phase crystals, multiphase composites with heterostructures demonstrate an increased propensity for interfacial polarization, which is attributed to the electron localization process caused by the larger WF disparity at heterointerfaces.^[^ ^20^ ^]^ Scholars commonly employ interfacial engineering to design heterojunctions with pronounced WF differences, optimizing microwave absorption performance through enhanced interfacial polarization. Researchers carefully designed the FeS and MoS_2_ heterointerfaces (with WF of 4.57 and 5.37 eV, respectively), and they showed that this large difference in WF significantly improved the interface polarization behavior.^[^ ^21^ ^]^ Therefore, WF provides theoretical evidence for interfacial polarization studies and is considered a valid descriptor for the interfacial polarization intensity, which has been extensively employed to support the elucidation of microwave absorption mechanisms. It is worth noting that according to current academic consensus, there is no universal or precise numerical value that can be defined as the threshold for triggering interfacial polarization between different materials. This is because interfacial polarization depends not only on the difference in work functions but also on various other factors. These include the intrinsic band structure of the composite materials (such as semiconductor/metal properties), the type of interface contact (chemical bonding or physical contact), and the testing frequency. Therefore, to determine whether interfacial polarization occurs, it is more meaningful to rely on experimental evidence and correlation analysis of overall performance, rather than on isolated work function differences.

### 2.4 Analyzing Electron Transfer, Electron Localization, and Polarization Behavior via Differential Charge Density (DCD), Electron Localization Function (ELF), and Bader Charge

1) DCD calculation serves as an essential tool for unraveling interatomic charge transfer dynamics by mapping electronic structure variations between composite systems and their original independent components. This methodology is particularly useful for studying polarization behavior in MAM, as it enables the systematic characterization of charge‐state modifications resulting from structural defects, elemental doping, single‐atom configurations, or heterojunction formation. The spatial visualization of charge distribution asymmetries obtained through DCD provides a clear understanding of energy dissipation pathways at the atomic scale.^[^ ^22^ ^]^ Notably, the emergence of calculated charge‐enriched or charge‐depleted zones can significantly influence dielectric properties through two primary mechanisms: the generation of oscillating dipoles via asymmetric charge polarization and the enhancement of carrier separation efficiency by establishing built‐in electric fields. 2) The ELF quantifies the degree of electron localization in space through analysis of electron density and kinetic energy. An ELF value approaching 1 signifies highly localized electronic configurations such as covalent bonds or lone pairs, these localized electrons struggle to achieve long‐range migration under alternating electromagnetic fields, thus contributing little to conductivity. While ELF values near 0.5 reflect free‐electron gas‐like delocalization characteristic of metallic bonding, these delocalized electrons can migrate under the influence of an external electric field, thereby enhancing conductivity. Values approaching zero correspond to completely delocalized electronic distributions observed in ionically bonded charge transfer regions.^[^ ^23^ ^]^ In addition, the discontinuous distribution of ELF values is the key to inducing polarization behavior. This discontinuity means that many dipoles are generated at the interface. In an alternating electromagnetic field, these dipoles will undergo rearrangement and relaxation processes, resulting in polarization loss. ELF maps provide an essential basis for understanding the relationship between electronic structure and material properties. 3) Bader charge calculations can transform the charge distribution characteristics into measurable metrics, enabling the precise quantification of charge transfer around specific atoms, functional groups, or structural domains. As a key visualization tool, Bader charge allows researchers to clarify how different structural modifications affect local charge states, thereby facilitating in‐depth analysis of EM loss mechanisms. As shown in Figure 1e, the calculated DCD, ELF maps, and Bader charge of the Ni‐N_4_ single‐atom model indicate that electron transfer from Ni atoms to the surrounding N atoms promotes the separation of positive and negative charges. This may lead to the formation of electron localization, which in turn enhances the polarization behavior of the material. Additionally, the researchers constructed computational models of Co_9_S_8_ (2 2 0) and Ni_9_S_8_ (1 1 4) faces, and used DCD and Bader charge analysis to demonstrate and quantify charge transfer at the heterointerface, thereby explaining the polarization behavior occurring within the material.^[^ ^24^ ^]^ This further confirms the effectiveness of DFT calculations in analyzing microscopic loss mechanisms.

## 3 Limitations and Mitigation Strategies of DFT in MAM Research

Although DFT provides insights into microwave loss mechanisms at the microscopic scale, its limitations in specific material systems cannot be ignored. These limitations and current mitigation strategies are listed below to properly understand the role that DFT plays in the research of MAM.

### 3.1 Deviation between Doping/Defect Models and Actual Materials

In the investigation of atomic doping or defect systems within MAM using DFT, a significant restriction arises from the fundamental differences between theoretical model scales and actual materials. To accurately simulate low‐concentration doping or defect scenarios, it is theoretically necessary to construct supercell models containing hundreds or even thousands of atoms, aiming to approximate the real chemical environment and long‐range interactions within acceptable statistical error margins. However, researchers are often limited by computational resources and time constraints, and must therefore employ simplified models based on smaller supercells containing only tens of atoms. This significant reduction in model size inevitably introduces systematic biases: such small‐scale models underestimate the average interatomic distances in the system, thus making it challenging to accurately compute key microscopic properties such as charge distribution.

To enhance the ability of small‐sized models to substitute for real materials while maintaining computational feasibility, researchers can employ a statistical sampling strategy. Specifically, this method involves designing small‐sized supercells with multiple atomic configurations to cover the local environmental diversity of doping atoms. And after performing multi‐threaded DFT calculations, all results are statistically averaged to effectively reduce systematic bias.^[^ ^25^ ^]^ However, this method relies on the assumption that doping/defect distributions are thermodynamically driven, but in actual materials may be kinetically controlled. For instance, defects formed during high‐temperature sintering may preferentially occupy grain boundaries rather than distribute randomly, but statistical methods are difficult to reflect this trend. In addition, exhaustively enumerating an exponential number of models poses a serious challenge to computational resources.

### 3.2 Challenges in DFT Calculation under Alternating EM Field

DFT, as a computational approach based on ground‐state energy principles, fundamentally aims to determine the static or quasi‐static electronic structure of many‐body systems. However, accurately simulating dynamic responses under alternating EM fields requires capturing the temporal evolution of internal electronic behavior, which lies beyond the theoretical scope of standard DFT. That is, it is difficult to create realistic radiation scenarios in DFT calculations of almost all material systems, let alone EM wave absorption processes at specific frequencies and powers, and the corresponding charge carrier behavior cannot be directly simulated.

Time‐dependent density functional theory (TD‐DFT) offers a promising approach for computing the electronic states of materials under time‐varying electromagnetic (EM) fields. By incorporating the potential energy term of an external EM field into the Kohn‐Sham equations, TD‐DFT can simulate the time evolution of electronic properties for a given atomic configuration.^[^ ^26^ ^]^ Nevertheless, the current TD‐DFT is applicable to ultrahigh frequency EM waves in the visible to UV bands (10^14^–10^16^ Hz), where the period of these processes is at the femtosecond level (10^−15^ s). The large difference in time scales prevents TD‐DFT from accurately interpreting nonlinear interactions between microwaves (ns, 10^−9^ s) and materials, leading to bias in predicting the electronic states of MAM. There is still a lack of suitable numerical computational methods to overcome the exponential scale difference, so TD‐DFT can only be used as a transitional strategy, and future research needs to further develop more accurate computational methods.

### 3.3 Electronic Structure Errors in Strongly Correlated Systems

The theoretical basis of DFT relies on the single‐electron approximation, where the many‐body electronic problem is simplified by assuming independent electron motion within an effective mean‐field potential. However, this approach exhibits intrinsic limitations in strongly correlated systems (such as Mott insulators, rare earth intermetallic compounds, and 2D electron gas), as large Coulombic electron‐electron interactions invalidate the single‐electron model, and collective excitations need to be considered instead.^[^ ^27^ ^]^ In the study of MAM, the EM properties of materials are highly dependent on the electronic structure and spin‐orbit coupling states, and the calculation of these parameters by DFT in strongly correlated systems is biased. For example, DFT may incorrectly predict the band structure of Mott insulators (e.g., NiO) as metallic states or narrow gap semiconducting, thus misleading the theoretical analysis of microwave attenuation mechanisms.

To mitigate the limitations of DFT in strongly correlated systems, the DFT+U approach incorporates the Hubbard U parameter, which corrects the Coulomb interaction for localized electrons in transition metal *d*‐orbitals and rare‐earth *f*‐orbitals. This enhancement results in calculated electronic band structures and other parameters that align more closely with experimental data. However, the accuracy of DFT+U calculations critically depends on the choice of the Hubbard U parameter, the selection of which remains largely subjective and semi‐empirical, lacking detailed guidelines.

## 4 Conclusion and Outlook

In short, this perspective critically assessed the utilization of DFT and its analytical paradigm in the field of microwave absorption, emphasizing its distinct utility while also describing the inherent constraints under particular conditions. Although DFT calculations have significantly advanced the development of MAM, they reveal limitations when addressing more complex material systems and underlying microscopic mechanisms. Recently, the development of AI has offered novel pathways to overcome these barriers. In order to promote DFT research of microwave absorbing to an intelligent and efficient stage, it is essential to discuss the future development trends of AI technology in DFT calculation.

### 4.1 Integrating Active Learning (AL) and Embedded Neural Networks (ENNs) to Overcome Sampling and Complexity Barriers in DFT Calculation

Regarding the physical assumptions and computational complexity issues in statistical sampling, we believe that the core AI technology for future research lies in the intelligent computational loop formed by AL and ENNs.

**Figure 2:**
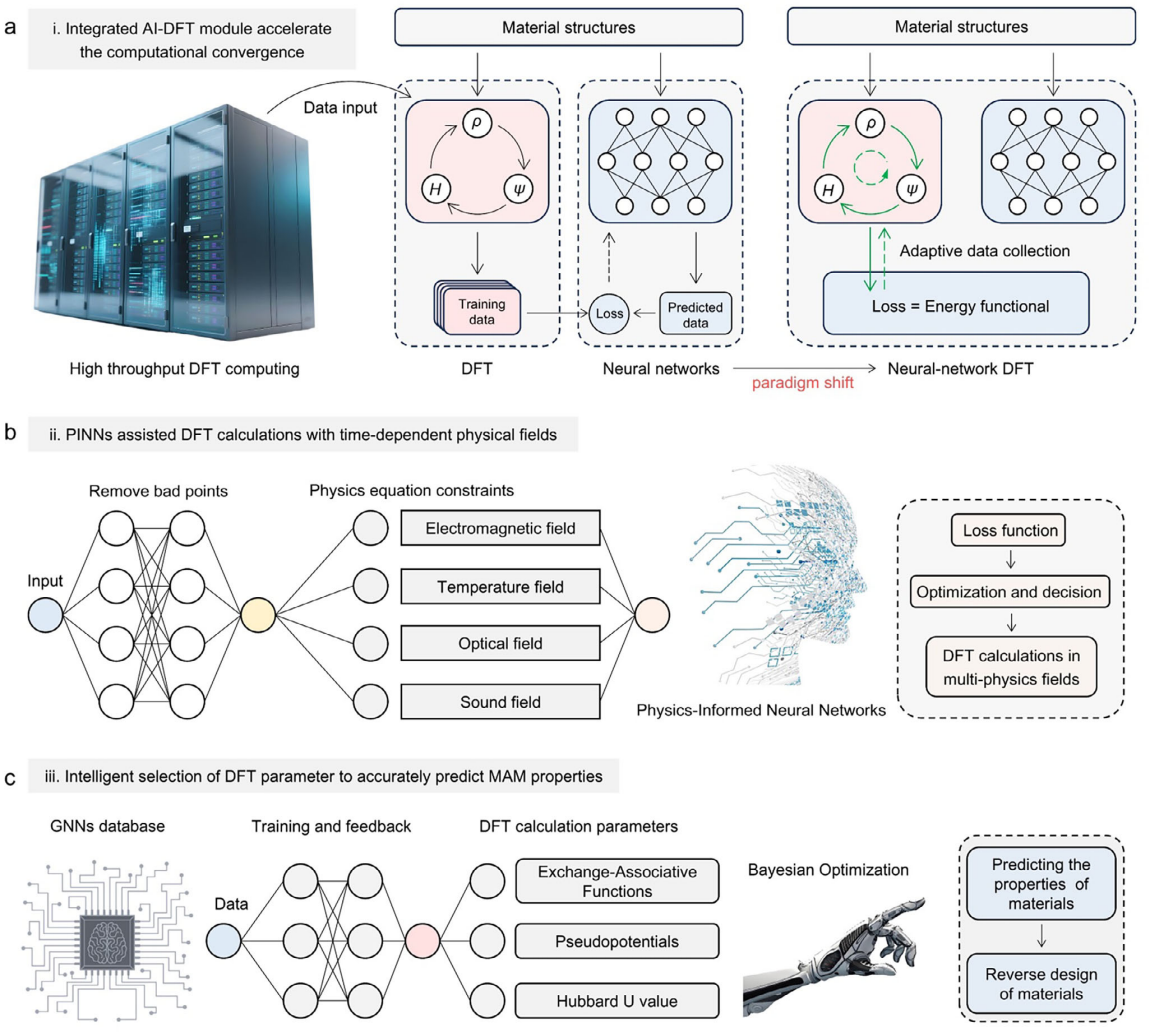
a) The differences between traditional deep learning and future neural‐network DFT algorithms; b) PINNs architecture for assisting the development of a multi‐physics DFT calculation module; c) Neural network and Bayesian optimization architecture for guiding DFT calculation parameter selection based on GNNs database.

On the one hand, AL techniques can quantify the information richness of different configurations by constructing numerous surrogate models. They then intelligently select the configurations with the highest information content for DFT calculations using a collection function and iteratively updating the models. This data‐driven approach captures the non‐equilibrium distribution governed by dynamics, thereby overcoming reliance on thermodynamic equilibrium assumptions and avoiding exhaustive enumeration of configurations. On the other hand, the current data‐driven supervised learning methodologies generate training data by conducting DFT calculations on diverse material structures, and then by designing a neural network so as to predict the DFT outputs. In this process, the neural network is separate from the DFT calculation. In future research, the integrated module of neural network and DFT will go beyond current methodologies. By embedding neural networks directly into the Self‐Concordant Field (SCF) iterative process, this module will quickly predict the Hamiltonian matrix of the system, thus avoiding the tedious basis function expansion step in the ab initio computation, and improving the convergence speed by several orders of magnitude. It also autonomously excludes bad data points through dynamic anomaly detection to guarantee the computational accuracy (**Figure** **2**a). The next‐generation module will fuse the rigor of quantum mechanics with the agility of AI technology to further unlock unprecedented insights into the electronic structure of matter.

### 4.2 Physics‐Informed Neural Networks (PINNs) Operators for Predicting Material Response under Time‐Dependent Physical Fields

Traditional DFT is limited to describing ground states or excited states under the adiabatic approximation and fails to calculate the electronic responses under real‐time physical field perturbations. The emergence of PINNs presents a promising methodology for breaking this dilemma.^[^ ^28^, ^29^ ^]^

Specifically, in future studies, researchers can encode physical laws (such as Maxwell's equations under time‐varying electromagnetic fields) as constraints into neural network architectures to construct a physically consistent constitutive model. The goal of this model is not to directly simulate electronic dynamics, but to establish functional relationships between the high‐frequency electronic structure and the low‐frequency electronic structure of materials. Its innovation lies in the ability to extrapolate from limited ultrahigh‐frequency DFT data to microwave bands through physical constraints, thereby bypassing the time scale gap faced by TD‐DFT direct solution and providing an alternative path for calculating the response of materials under real microwave irradiation. Based on this, a database can be established, and Fourier neural operators are used to learn solution operators from external field conditions to system responses, predicting dynamic behavior under different microwave fields almost instantaneously, providing a new paradigm for simulating nonlinear interactions between microwaves and materials (Figure 2b).

### 4.3 Graph Neural Networks (GNNs) and Bayesian Optimization for DFT Calculation in Strongly Correlated Systems

The current DFT+U method used in strongly correlated systems relies on the empirical selection of the Hubbard U value. Future research may focus on utilizing GNNs and Bayesian optimization techniques to bypass or modify the single‐electron approximations of DFT in a data‐driven manner, replacing traditional trial‐and‐error paradigms.

The message transmission mechanism of GNNs is to transmit the crystal structure in the form of a graph, which can naturally and accurately describe the local interactions between atoms, thus directly learning the end‐to‐end complex mapping from atomic configuration to final electronic properties.^[^ ^30^ ^]^ It no longer relies on the single electron approximation or Hubbard U value, but directly establishes the structure performance relationship, fundamentally avoiding the systematic bias of DFT in strongly correlated systems. If the DFT+U paradigm remains necessary, we propose employing a Bayesian optimization workflow to automatically and objectively determine the Hubbard U value.^[^ ^31^ ^]^ Specifically, we can treat the U value as an optimization variable and use the agreement between DFT+U calculations and experimental benchmarks as the objective function. Based on this, Bayesian optimization intelligently proposes the U value for the next calculation, finding the optimal U value that best matches calculations with experiments in the fewest iterations. This transforms the U selection from subjective experience into a systematic, data‐driven optimization problem, significantly enhancing the predictive reliability of DFT+U calculations.

## Conflict of Interest

The authors declare no conflict of interest.

## References

[1] M. Liu , L. Yang , Z. Wu , G. Chen , X. Wang , X. Yang , G. Liang , R. Che , Nat. Commun. 2025, 16, 5633. 40595586 10.1038/s41467-025-60890-3 PMC12219658

[2] R. Zhang , B. Yuan , F. Pan , H. Liang , H. Jiang , H. Guo , Y. Rao , S. Zheng , L. Ruan , C. Wu , Y. Yang , W. Lu , Matter 2025, 8, 101956.

[3] K. Zhang , Y. Liu , X. Li , X. Wang , J. Liu , X. Liu , Adv. Mater. 2025, 37, 2506386. 10.1002/adma.202506386 40519094

[4] N. Qu , H. Sun , Y. Sun , M. He , R. Xing , J. Kong , Nat. Commun. 2024, 15, 5642. 38969643 10.1038/s41467-024-49762-4 PMC11226717

[5] Z. Wang , Z. Li , B. Li , A. Shi , L. Zhang , Y. Zhu , F. Ye , S. Yu , Adv. Mater. 2024, 36, 2412605. 10.1002/adma.202412605 39428894

[6] M. Qin , L. Zhang , H. Wu , Adv. Sci. 2022, 9, 2105553. 10.1002/advs.202105553 PMC8981909 35128836

[7] J. Tao , Y. Yan , J. Zhou , J. Wang , P. Chen , R. Tan , L. Xu , H. Zhu , W. Zhu , H. Huang , X. Tao , Z. Yao , Nat. Commun. 2025, 16, 3163. 40175363 10.1038/s41467-025-58448-4 PMC11965476

[8] L. Zhou , P. Hu , M. Bai , N. leng , B. Cai , H. Peng , P. Zhao , Y. Guo , M. He , G. Wang , J. Gu , Adv. Mater. 2025, 37, 2418321 10.1002/adma.202418321 39726342

[9] L. Rao , M. Huang , X. Wang , Y. Qian , Z. Yan , L. Wang , Q. Li , R. Che , Angew. Chem. 2024, 137, 202418338. 10.1002/anie.202418338 39472277

[10] F. Gu , W. Wang , H. Meng , Y. Liu , L. Zhuang , H. Yu , Y. Chu , Matter 2025, 8, 102004.

[11] L. Yan , X. Li , Y. Zhang , D. Kim , Y. Zhang , Z. Zhang , P. Ying , F. Xu , C. Liu , Z. Zeng , Acta Mater. 2025, 294, 121154.

[12] R. Che , Z. Wu , B. Quan , R. Zhang , H. Zhang , J. Zhang , W. Lu , Adv. Funct. Mater. 2023, 33, 2303108.

[13] W. Acuna , W. Wu , J. Bork , M. Doty , M. Jungfleisch , L. Gundlach , J. Zide , Adv. Funct. Mater. 2024, 34, 2401853.

[14] J. Wen , L. Deng , H. Shen , Q. Chen , H. Wu , Adv. Funct. Mater. 2025, 10.1002/adfm.202519086.

[15] L. Qiu , C. Zeng , X. Feng , L. Zhuang , W. Liu , Z. Fu , Appl. Phys. Lett. 2025, 126, 083101.

[16] S. Hui , X. Zhou , L. Zhang , H. Wu , Adv. Sci. 2024, 11, 2307649. 10.1002/advs.202307649 PMC10853738 38044282

[17] J. Wen , G. Chen , S. Hui , Z. Li , J. Yun , X. Fan , L. Zhang , Q. He , X. Liu , H. Wu , Adv. Powder Mater. 2024, 3, 100180.

[18] C. Zhang , J. Jiang , Z. Guan , Y. Zhang , Y. Li , B. Song , W. Shao , L. Zhen , Adv. Sci. 2024, 11, 2306159. 10.1002/advs.202306159 PMC10939080 38044305

[19] H. Xu , M. Liu , L. Huang , X. Zhang , Z. Ma , C. Zhu , F. Cao , Y. Chen , Adv. Funct. Mater. 2025, 35, 2502952.

[20] S. Hui , Q. Chen , K. Tao , L. Zhang , X. Fan , R. Che , H. Wu , Adv. Mater. 2025, 37, 2415844. 10.1002/adma.202415844 39593259

[21] Y. Shen , Z. Ma , F. Yan , C. Zhu , X. Zhang , Y. Chen , Adv. Funct. Mater. 2025, 35, 2423947.

[22] S. Cheng , D. Sheng , S. Mukherjee , Y. Huang , R. Cao , A. Xie , W. Dong , R. Fischer , W. Li , Nat. Commun. 2024, 15, 9077. 39433804 10.1038/s41467-024-53465-1 PMC11494010

[23] J. Wen , Y. Liu , S. Hui , L. Deng , L. Zhang , X. Fan , Q. Chen , X. Liu , X. Li , N. Yan , H. Wu , Matter 2025, 8, 102151.

[24] J. Liu , S. Zhang , D. Qu , X. Zhou , M. Yin , C. Wang , X. Zhang , S. Li , P. Zhang , Y. Zhou , K. Tao , M. Li , B. Wei , H. Wu , Nano‐Micro Lett. 2025, 17, 24. 10.1007/s40820-024-01515-0 PMC11436618 39331290

[25] J. Li , F. Pan , G. Zhang , Z. Liu , H. Dong , D. Wang , Z. Jiang , W. Ren , Z. Ye , M. Todorovic , P. Rinke , Small Struct. 2024, 5, 2400268.

[26] M. Yan , X. Han , C. Zhang , Water Res. 2017, 496, 124, 496e503. 28802134 10.1016/j.watres.2017.08.004

[27] M. Hepting , D. Li , C. Jia , H. Lu , E. Paris , Y. Tseng , X. Feng , M. Osada , E. Been , Y. Hikita , Y. Chuang , Z. Hussain , K. Zhou , A. Nag , M. Fernandez , M. Rossi , H. Huang , D. Huang , Z. Shen , T. Schmitt , H. Hwang , B. Moritz , J. Zaanen , T. Devereaux , W. Lee , Nat. Mater. 2020, 19, 381. 31959951 10.1038/s41563-019-0585-z

[28] X. Gong , W. Duan , H. Li , N. Zou , R. Xu , Y. Xu , Nat. Commun. 2023, 14, 2848. 37208320 10.1038/s41467-023-38468-8 PMC10199065

[29] Z. Chen , Y. Liu , H. Sun , Nat. Commun. 2021, 12, 6136. 34675223 10.1038/s41467-021-26434-1 PMC8531004

[30] G. Corso , H. Stark , S. Jegelka , T. Jaakkola , R. Barzilay , Nat. Rev. Method Primers 2024, 4, 17.

[31] Y. Zuo , M. Qin , C. Chen , W. Ye , X. Li , J. Luo , S. Ong , Mater. Today 2021, 51, 126.

